# Species-Specific Effects of Epigeic Earthworms on Microbial Community Structure during First Stages of Decomposition of Organic Matter

**DOI:** 10.1371/journal.pone.0031895

**Published:** 2012-02-21

**Authors:** María Gómez-Brandón, Marta Lores, Jorge Domínguez

**Affiliations:** 1 Departamento de Ecoloxía e Bioloxía Animal, Facultade de Bioloxía, Universidade de Vigo, Vigo, Spain; 2 University of Innsbruck, Institute of Microbiology, Innsbruck, Austria; 3 Laboratorio de Investigación y Desarrollo de Soluciones Analíticas. Departamento de Química Analítica, Facultad de Química, Santiago de Compostela, Spain; Auburn University, United States of America

## Abstract

**Background:**

Epigeic earthworms are key organisms in organic matter decomposition because of the interactions they establish with microorganisms. The earthworm species and the quality and/or substrate availability are expected to be major factors influencing the outcome of these interactions. Here we tested whether and to what extent the epigeic earthworms *Eisenia andrei*, *Eisenia fetida and Perionyx excavatus*, widely used in vermicomposting, are capable of altering the microbiological properties of fresh organic matter in the short-term. We also questioned if the earthworm-induced modifications to the microbial communities are dependent on the type of substrate ingested.

**Methodology/Principal Findings:**

To address these questions we determined the microbial community structure (phospholipid fatty acid profiles) and microbial activity (basal respiration and microbial growth rates) of three types of animal manure (cow, horse and rabbit) that differed in microbial composition, after being processed by each species of earthworm for one month. No differences were found between earthworm-worked samples with regards to microbial community structure, irrespective of type of manure, which suggests the existence of a bottleneck effect of worm digestion on microbial populations of the original material consumed. Moreover, in mesocosms containing cow manure the presence of *E. andrei* resulted not only in a decrease in bacterial and fungal biomass, but also in a reduced bacterial growth rate and total microbial activity, while no such reduction was found with *E. fetida* and *P. excavatus*.

**Conclusions/Significance:**

Our results point to the species of earthworm with its associated gut microbiota as a strong determinant of the process shaping the structure of microbial communities in the short-term. This must nonetheless be weighed against the fact that further knowledge is necessary to evaluate whether the changes in the composition of microbiota in response to the earthworm species is accompanied by a change in the microbial community diversity and/or function.

## Introduction

The aboveground net primary production, around 80–90%, enters the soil food web as dead plant material in most terrestrial ecosystems [1]. Bacteria and fungi, which form up to 90% of the soil microbial biomass, are the primary litter decomposers [2], but decomposer food webs are highly complex and include protozoa, nematodes, microarthropods and earthworms, among others [3]. When present, earthworms are known to play a key role in organic matter decomposition, thus significantly accelerating decomposition rates and nutrient turnover [4]. Earthworms may affect the decomposition of organic matter through gut associated processes (direct effects), i.e. via the effects of ingestion, digestion and assimilation of the organic matter and microorganisms, which are then released in earthworm casts [5], [6]; and cast associated processes (indirect effects) that are more closely associated with the presence of unworked material and to physical modification of the egested material [7]. Such indirect effects are derived from direct effects, and include processes such as the ageing of earthworm-worked material (weeks to months), and mixing of such material with substrates that have not been processed by earthworms yet [8]. According to this rationale, it is difficult to separate direct and indirect processes and their components, because they occur simultaneously in time and space.

Vermicomposting is an example of an enhanced decomposition process, in which both gut and cast associated processes play a key role in determining the characteristics of the microbial decomposer communities [9]. Vermicomposting systems sustain a complex food web [10], in which detritivore earthworms interact intensively with microorganisms and other fauna within the decomposer community, accelerating the stabilization of organic matter and greatly modifying its physical and biochemical properties [9]. More specifically, epigeic earthworms may affect microbial decomposer activity by grazing directly on microorganisms, and by increasing the surface area available for microbial attack after comminution of organic matter [11]. These activities enhance the turnover rate and productivity of microbial communities, thereby increasing the rate of decomposition. These earthworm species may also affect other fauna directly, mainly through the ingestion of microfaunal groups (protozoa and nematodes) that are present within the organic detritus consumed [5], or indirectly, modifying the availability of resources for these groups [12]. Furthermore, epigeic earthworms are known to excrete large amounts of holorganic casts, which are difficult to separate from the ingestible substrate [9]. The contact between these different processed materials may thus affect the decomposition rates, due to the presence of microbial populations in earthworm casts different from those contained in the material prior to ingestion [13], [14]. In addition, the nutrient content of the egested materials differs from that in the ingested material [15], which may enable better exploitation of resources, because of the presence of a pool of readily assimilable compounds in the casts. Indeed, Aira *et al.* [15] found an increase in the labile carbon pool of pig manure in a short-term experiment (72 h) with the epigeic earthworm *Eisenia fetida*; such effects were density-dependent. They detected greater values of dissolved organic carbon (DOC) with the highest density of earthworms (100 earthworms per mesocosm) than that in the control, whereas the low and medium earthworm densities (25 and 50 earthworms per mesocosm) showed intermediate values. The presence of and interaction between microbial communities from both worm-worked and unworked materials has been shown to improve decomposition rates by altering levels of the microbial community activity and enhancing nutrient release [8], as well as by modifying the functional diversity of microbial populations [16], which are key factors for organic matter decomposition. Aira *et al.* [16] observed an increase in the capabilities of the microbial populations of pig slurry to use more diverse carbon pools in a long-term experiment (36 weeks) with the epigeic earthworm *E. fetida*, which suggests that microbial communities use the available energy more efficiently in the presence of earthworms.

The vermicomposting process includes two different phases regarding earthworm activity: (i) an active phase during which earthworms process the organic substrate, thereby modifying its physical state and microbial composition, and (ii) a maturation phase marked by the displacement of the earthworms towards fresher layers of undigested substrate, during which the microorganisms take over the decomposition of the earthworm-processed substrate [16], [17]. The length of the maturation phase is not fixed, and depends on the efficiency with which the active phase of the process takes place, which in turn is largely determined by the composition of the parent material [9]. In this way, Aira & Domínguez [18] observed a reduction in microbial activity in the casts of the epigeic earthworm *E. fetida* fed with cow manure, whereas they did not detect any changes in this parameter when *E. fetida* was fed on pig slurry. Thus, the quality and/or substrate availability is expected to be a major factor influencing the interactions within the decomposer community during vermicomposting; but the outcome of these interactions may also depend on the earthworm species considered. Earthworms of different functional groups, or even different species within the same functional group, have a particular mode of food selection, ingestion, digestion, assimilation and movement, thus their importance in mixing, decomposition or nutrient release, as well as in the structure and activity of microbial communities will vary both qualitatively and quantitatively [19]. Up to now, previous studies dealing with the effects of epigeic earthworms on microorganisms have primarily been focused on evaluating separately, either the impact of the composition of parent material [14], [18], [20]–[23] or the earthworm species [5]–[6], [24]–[25] on the structure and activity of microbial communities. As such, to date little is yet known about the link between the above-mentioned factors (i.e., parent material and earthworm species) in order to evaluate which factor is the most determinant in the process with respect to the changes in the composition of microbial populations during vermicomposting. This may have important implications for the optimization of this process and contribute in better understanding the relationships between epigeic earthworms and microorganisms during the decomposition of organic matter.

The aim of the present study was therefore to investigate whether and to what extent the epigeic earthworm species *Eisenia andrei*, *Eisenia fetida and Perionyx excavatus*, widely used for processing organic substrates [9], are capable of altering the microbiological properties of fresh organic matter during the active phase of vermicomposting. Moreover, we also questioned if the earthworm species -induced modifications to the microbial communities are dependent on the type of substrate ingested. To address these questions we investigated the impact of these earthworm species on the microbial community structure (phospholipid fatty acid profiles) and microbial activity (basal respiration and microbial growth rates) of three types of animal manure (cow, horse and rabbit manure), which differed in microbial composition. We also studied how such changes in microbial communities affected the rate of organic matter decomposition, by analysing the loss of carbon as a result of earthworm activity.

## Results

**Figure 1:**
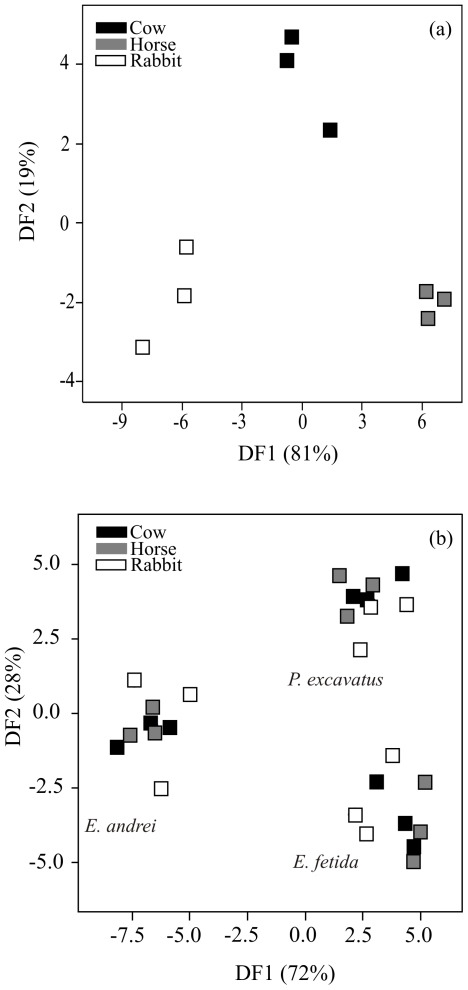
Discriminant plot for the first and second functions of the PLFAs identified in (a) the three raw animal manures (cow, horse and rabbit), and (b) the manures after being processed by the epigeic earthworm species *Eisenia andrei*, *Eisenia fetida* and *Perionyx excavatus* during the active phase of vermicomposting.

Microbial communities in the three raw manures (cow, horse and rabbit) were clearly differentiated from each other (Fig. 1a), as shown by the discriminant analysis of the twenty-five identified PLFAs (i14:0, 14:0, i15:0, a15:0, 15:0, i16:0, 16:1ω9, 16:1ω7, 16:1ω5, 16:0, 10Me16:0, i17:0, a17:0, cy17:0, 17:0, 10Me17:0, 18:2ω6,9, 18:1ω9, 18:1ω7, 18:0, 10Me18:0, cy19:0, 20:4ω6, 20:5ω3, 20:3ω6). Moreover, epigeic earthworms played a key role in shaping the structure of microbial communities after the active phase of vermicomposting. We found that the shifts in phospholipid fatty acid (PLFA) profiles were strongly influenced by the species of earthworm (Fig. 1b), irrespective of the type of manure (ANOVA F_4,18_ = 0.37, *P* = 0.83). The first discriminant function contributed in differentiating the substrates processed by *E. andrei* from those processed by *E. fetida* and *P. excavatus* (Fig. 1b), accounting for 72% of the variance (ANOVA F_2,18_ = 57.38, *P*<0.001), whereas the second function (accounting for 28% of the variance) mainly separated the substrates processed by *E. fetida* from those processed by *P. excavatus* (ANOVA F_2,18_ = 62.47, *P*<0.001; Fig. 1b).

**Figure 2:**
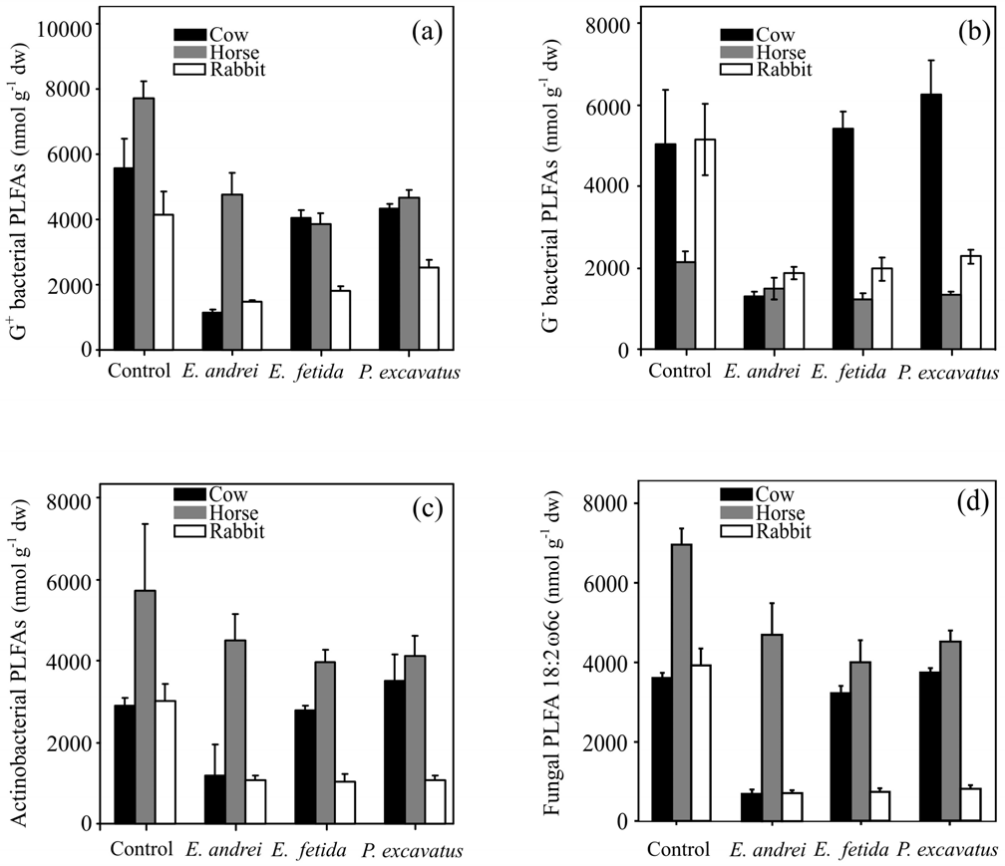
Changes in (a) Gram-positive bacterial PLFAs, (b) Gram-negative bacterial PLFAs, (c) actinobacterial PLFAs, and (d) fungal PLFA 18:2ω6c of the three different animal manures (cow, horse and rabbit) after being processed by the epigeic earthworm species *Eisenia andrei*, *Eisenia fetida* and *Perionyx excavatus* during the active phase of vermicomposting. Values are means ± SE. Controls are the manures incubated without earthworms.

The activity of *E. andrei* drastically reduced the concentration of both Gram-positive and Gram-negative bacterial PLFAs in cow manure relative to the control (4.8 and 3.9 times, respectively), while no such pronounced decreases were detected in relation to the activity of *E. fetida* and *P. excavatus* (Fig. 2a,b). However, in the treatment with horse manure, the abundance of both G+ and G− bacterial PLFAs was approximately 1.8 and 1.6 times lower than that in the control, and were similar for the three earthworm species (Fig. 2a,b). In the mesocosms with rabbit manure, the activity of the three earthworm species reduced the abundance of those microbial groups to a greater extent than in horse manure (2.2 and 2.5 times lower respectively; Fig. 2a,b), resulting in a significant interaction between the type of manure and earthworm activity (G^+^ bacteria: ANOVA, F_6,24_ = 4.02, *P*<0.01; G^−^ bacteria: ANOVA, F_6,24_ = 6.5, *P*<0.001). A decrease in the abundance of actinobacterial PLFAs was also observed with earthworm presence (ANOVA, F_3,24_ = 3.7, *P*<0.05; Fig. 2c), being 1.4 and 2.8 times lower relative to the control in the presence of the three epigeic earthworms feeding on horse and rabbit manure respectively (Fig. 2c). However, as occurred with G^+^ and G^−^ bacteria, in mesocosms containing cow manure such reduction in actinobacterial PLFAs was only detected with *E. andrei* (2.5 times lower, Fig. 2c). Accordingly, a decrease in fungal biomass, as assessed by the PLFA biomarker 18:2ω6c, was observed in mesocosms of *E. andrei* fed with cow manure (5.3 times lower; Fig. 2d), while no such reduction was found in relation to the activity of *E. fetida* and *P. excavatus* (Fig. 2d). However, in the treatments with horse and rabbit manure, the fungal biomass was two and six times lower than that in the control (Fig. 2d), producing a significant interaction between the type of manure and earthworm activity (ANOVA, F_6,24_ = 7.9, *P*<0.001).

**Figure 3:**
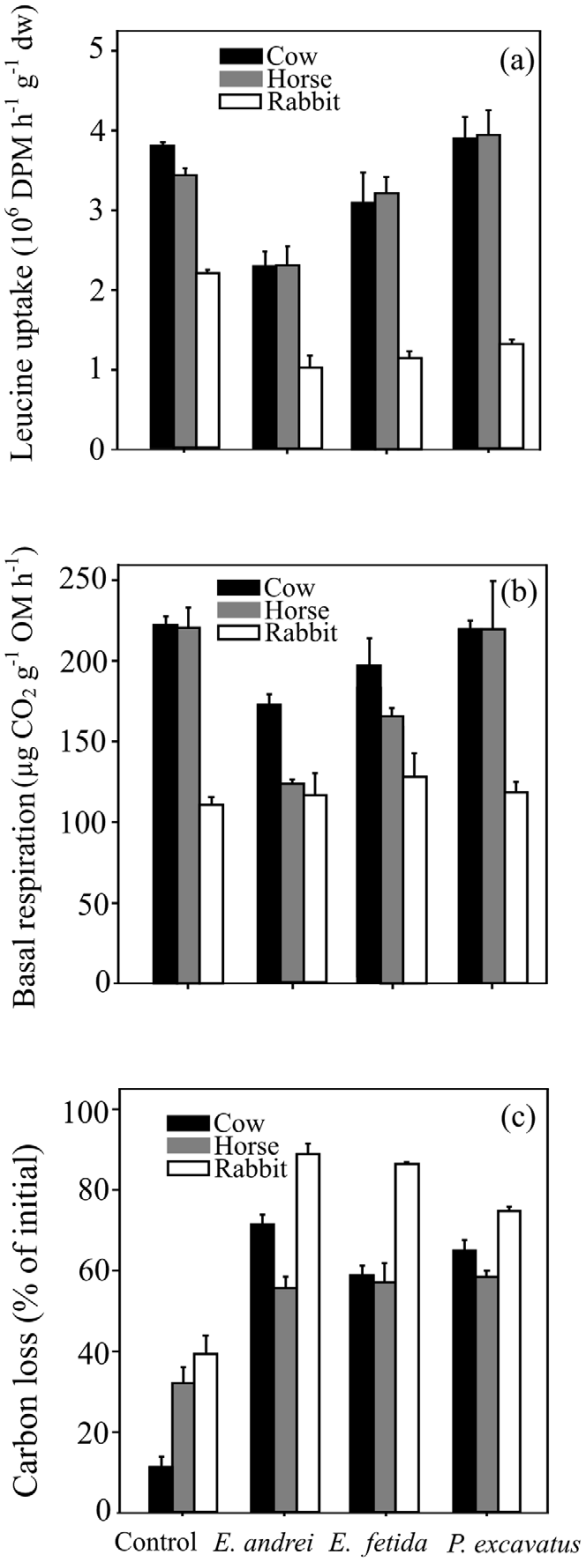
Changes in (a) bacterial growth rate, estimated as leucine uptake, (b) microbial activity, measured as basal respiration, and (c) carbon loss (percentage of initial) of the three different animal manures (cow, horse and rabbit) after being processed by the epigeic earthworm species *Eisenia andrei*, *Eisenia fetida* and *Perionyx excavatus* during the active phase of vermicomposting. Values are means ± SE. Controls are the manures incubated without earthworms.

The earthworm activity greatly decreased the bacterial growth rate, estimated by the incorporation of ^3^H-leucine into proteins, after the active phase of vermicomposting; although this effect varied depending on the type of manure (ANOVA F_6,24_ = 3.61, *P*<0.05; Fig. 3a). In mesocosms with cow and horse manures, *E. andrei* reduced the bacterial growth rate by approximately 1.5 times relative to the control without earthworms; no significant differences were detected with *E. fetida* and *P. excavatus* (Fig. 3a). However, in rabbit manure, the bacterial growth rate was about two times lower in the presence of the three earthworm species than in the control (Fig. 3a). Despite the consistent effects on bacterial growth, earthworm activity did not affect fungal growth rate (data not shown), irrespective of the type of manure (ANOVA F_6,24_ = 0.70, *P* = 0.65). The changes in microbial activity measured as basal respiration depended on the type of manure (ANOVA F_6,24_ = 4.90, *P*<0.01; Fig. 3b). In mesocosms containing cow and horse manures, the pattern of microbial activity was similar to the pattern of bacterial growth (Fig. 3b). However, no significant changes in microbial activity were found in rabbit manure after it was processed by any of the three epigeic earthworm species (Fig. 3b). The activity of earthworms accelerated the loss of total carbon relative to the control after the active phase of vermicomposting (ANOVA, F_3,24_ = 153.7, *P*<0.001; Fig. 3c); this effect was more pronounced in cow manure (six times greater) than in the horse and rabbit manures (around two times greater) (Fig. 3c).

## Discussion

Several studies have already shown changes in the structure of microbial communities assessed by PLFA profiles in the presence of epigeic earthworms [17], [23], [26]–[27]. However, the present results are unique in that although the three types of animal manure differ in their microbial composition, there were no differences between earthworm-worked samples derived from the different types of manure after they had been processed by each earthworm species with regards to microbial community structure. Similar results were also reported with fatty acid methyl ester (FAMES) profiles [28]. They found similar structures of microbial communities in three different animal manures (cow, horse and pig manure) after having been processed by the earthworm species *Eisenia andrei*, *Eudrilus eugeniae* and *Lumbricus rubellus* for one month. The previous and the present study together are thus consistent with recent results that provide strong evidence for a bottleneck effect of worm digestion (*E. andrei*) on microbial populations of the original material consumed [23]. They found no differences between fresh cast samples derived from different types of manure with respect to microbial community structure, which suggests that the direct effects of these earthworm species on the microbial community composition are largely determined by factors other than the parent material. These latter authors [23] point to the earthworm gut as a major shaper of microbial communities, acting as a selective filter for microorganisms contained in the substrate, thereby favouring the existence of a microbial community specialized in metabolizing compounds produced or released by the earthworms, in the egested materials. Accordingly, specific microbial groups have been shown to respond differently to the gut environment [29] and selective effects on the presence and abundance of microorganisms during the passage of organic material through the gut of epigeic earthworms have been observed. For instance, some bacteria are activated during passage through the gut, whereas others remain unaffected and others are digested in the intestinal tract and thus decrease in number [30]–[32]. The selective effects on ingested microbes through the earthworm gut may be caused by competitive interactions between those ingested and the endosymbiotic microbes that reside in the gut; and/or by selective suppressive activity of gut fluids against specific microbial groups [33]. Ultimately, the inoculation of those communities from worm-worked material (i.e., casts and/or vermicompost) in raw organic matter is expected to modify decomposition pathways, by altering microbial community levels of activity; these effects are also species-specific dependent [8].

The previous findings underscore the influence that the gut with its microbiota might have on the changes that occurred in the substrate as a result of earthworm activity. The gut is mainly responsible for extracting energy from ingested food and it is considered one of the most important metazoan organs [34]. There is an intimate interaction between gut bacteria, having the metabolic capacity to break down those energy sources the host cannot directly utilize, which indicates that the microbial profile of the gut is expected to be an important determinant of earthworm metabolism. The distribution pattern of the earthworm gut microbiota at the host population level and the factors that determine such pattern are therefore of fundamental importance to understand host-microbiota interactions and their implications in decomposition pathways [35]. Recently, Thakuria *et al.* [36] found evidences that the selection of the gut wall-associated bacteria in some earthworm species (the anecic *Lumbricus terrestris* and *L. friendi* and the endogeic *Aporrectodea caliginosa* and *A. longa*) is a process of natural selection, and the strongest determinants for this process are in the order of ecological group>habitat> earthworm species. Bearing in mind that different earthworm species within the same functional group can harbour distinct gut-wall associated microbiota, together with the fact that the earthworms used in the present study come from different stock cultures and no characterization of their gut microbiota was performed prior to the experiment, one might speculate that the differential effects of these earthworm species with regards to microbial community structure may reflect forces exerted by the different earthworm gut microbiota rather than earthworm species-specific effects *per se.*

Epigeic earthworms possess a diverse pool of digestive enzymes which enables them to digest bacteria, protozoa, fungi and partly decomposed plant debris [37]. Recent reports suggest that the digestion of organic material by these earthworm species has negative effects on microbial biomass [23], [32], [38]. In accordance with this, we found that bacterial and fungal populations decreased in the three types of animal manure with earthworm presence, although in cow manure such a decrease was only detected in the presence of *E. andrei*. More specifically, the activity of *E. andrei* reduced the abundance of G+ bacteria to a greater extent than G− bacteria in this type of manure (4.8 and 3.9 times respectively). These findings are consistent with the results of a previous study, in which we observed that the earthworm *E. andrei* had a more pronounced impact on G+ bacteria than G− bacteria through the gut associated processes [23]. Other previous studies involving the effects of epigeic earthworms on microorganisms have also shown that G− bacteria can survive the transit through the earthworm gut [39]–[41]. Such differences may be due to the fact that G− bacteria possess an outer membrane composed of lipopolysaccharides, which provides them with structural integrity increasing the negative charge of the cellular membrane and protecting them against certain types of chemical attack [42]. This decreasing trend on microbial biomass with earthworm presence appears to be permanent over time, since during the maturation of vermicompost bacterial and fungal biomass tends to be maintained at low values, which depend on the rate of detrital input [17]. Epigeic earthworms may have also reduced the abundance of these microbial groups indirectly by the depletion of the resources used by microbes, since greater losses of carbon were found as a result of earthworm activity after the active phase of vermicomposting.

Despite the consistent effects on total microbial activity and bacterial growth, earthworm activity did not affect the fungal growth rate, irrespective of the type of manure. Similarly, Aira *et al.* [15] detected a significant increase in the fungal biomass of pig manure, measured as ergosterol content, in a short-term experiment (72 h) with the earthworm species *E. fetida*, and the effect depended on the density of earthworms. A higher fungal biomass was found at intermediate and high densities of earthworms (50 and 100 earthworms per mesocosm, respectively), which suggests that there may be a threshold density of earthworms at which fungal growth is triggered. This priming effect on fungal populations was also observed in previous short-term experiments in the presence of the epigeic earthworms *Eudrilus eugeniae* and *Lumbricus rubellus* fed with pig and horse manure, respectively [28], [38]. The first stages of decomposition in animal manures are predominated by bacteria (around 70% of the total microbial biomass as assessed by PLFA analysis), because of the availability of water and readily decomposable compounds [9]. Fungi, which are mainly present as spores in this type of substrate, have been found to be more competitive during the maturation stage with regard to the degradation of more slowly decomposable compounds such as cellulose, hemicellulose and lignin. Aira *et al.* [43] reported an increase in the fungal biomass, measured as ergosterol content in a long-term experiment (36 weeks) with the epigeic earthworm *E. fetida*, reaching up 7.5 times more than in the control; this priming effect on fungal populations was accompanied by a higher rate of cellulose decomposition in the presence of earthworms. These contrasting effects on bacterial and fungal populations with earthworm activity are thus expected to have important implications on decomposition pathways during vermicomposting, because there exist important differences between both microbial decomposers related to resources requirements and exploitation. This is based on the fact that fungi can immobilize great quantities of nutrients in their hyphal networks, whereas bacteria have a more exploitative nutrient use strategy by rapidly using newly produced labile substrates [1].

Overall, the present study provides insight into the short-term effects of epigeic earthworms on the microbial decomposers, and further illustrates the important role of these earthworm species in shaping the structure of microbial communities during the active phase of vermicomposting. Having determined that the earthworm species with its associated gut microbiota was the strongest determinant of the process, it will be of future interest to evaluate whether the changes in the composition of microbiota in response to the earthworm species is accompanied by a change in the microbial community diversity and/or function. Ultimately, these earthworm-specific effects on microbial communities may have important implications for the production of plant container media and for impoverished and/or intensively fertilized soils, because by varying the earthworm species used in vermicomposting it is possible to obtain specific vermicomposts in relation to the structure of microbial communities, which can be used for different practical applications.

## Materials and Methods

### Ethics statement

No permits were required for the collection of animal manures as the farm in question provides the University of Vigo with manure free of charge.

### Experimental material and set up

Animal manures were collected from a farm near the University of Vigo (Galicia, NW Spain). The main chemical properties of the three types of animal manure are shown in Table 1. Specimens of *Eisenia andrei* and *Eisenia fetida* were sampled (hand-sorted method) from stock cultures reared under laboratory conditions (20±2°C). Specimens of *Perionyx excavatus* were obtained from a commercial supplier in Brazil (Minhobox).

**Table 1:** Main chemical properties of the three types of animal manure (cow, horse and rabbit) used in this study.

	Cow manure	Horse manure	Rabbit manure
pH	7.55±0.02	7.30±0.04	8.17±0.06
Electrical conductivity (mS cm^−2^)	0.17±0.02	0.13±0.01	0.23±0.02
Total C (mg g^−1^ dw)	400±38	446±39	353±27
Dissolved organic carbon (mg kg^−1^ dw)	36435±2617	24240±1027	21348±970
Total N (mg g^−1^ dw)	21±4	16±1	22±4
NH_4_^+^ (mg kg^−1^ dw)	9614±94	8991±66	4223±134
NO_3_^−^ (mg kg^−1^ dw)	172±21	183±14	397±61
C to N ratio	19±0.2	28±0.2	16±0.2

Values are means ± standard error (n = 3).

The mesocosms consisted of plastic containers (2 L) filled to three quarters of their capacity with sieved, moistened vermiculite and inoculated with 10 mature earthworms. Vermiculite is a hydrated silicate mineral resembling mica and does not contain any organic nutrients, which thus obliged the earthworms to ingest the substrate provided. A plastic mesh (5 mm pore size) was placed over the surface of the vermiculite permitting the mobility of earthworms from the vermiculite bedding to the substrate (200 g, fresh weight) that is placed on top of the mesh. The mesocosms were covered with perforated lids and stored in random positions in an incubation chamber at 20°C and 90% relative humidity. Control mesocosms consisted of each type of manure incubated without earthworms. Each treatment was replicated three times. The length of the active phase depends greatly on the rates at which the earthworms ingest and process the substrate [9]. The high rate of consumption, digestion and assimilation of organic matter by these earthworm species resulted in the substrates being completely processed by the earthworms in one month, as previously shown by Lores *et al.* [28]. After this time, the earthworms were removed from the mesocosms and the processed material was collected from the surface of the vermiculite. The same amount of sample was also collected from the control mesocosms. All samples were immediately stored at −20°C for phospholipid fatty acid (PLFA) analysis and at 4°C for determining microbial growth rates and total microbial activity, assessed by basal respiration.

### Analytical procedures

Electrical conductivity and pH were measured in aqueous extracts (1∶10, *w/v*). Total C and N contents were analysed in dried samples, in a Carlo Erba 1500 C/N analyser. Inorganic N (NH_4_^+^ and NO_3_^−^) was determined in 0.5 M K_2_SO_4_ extracts (1∶5, *w/v*) using a modified indophenol blue technique [44] with a Bio-Rad Microplate Reader 550. Dissolved organic carbon was determined colorimetrically in microplates after moist digestion (K_2_Cr_2_O_7_ and H_2_SO_4_) of aliquots of 0.5 M K_2_SO_4_ extracts.

Bacterial and fungal biomass was assessed by PLFA analysis. The sum of Gram-positive (i14:0, i15:0, a15:0, i16:0, i17:0 and a17:0); and Gram-negative bacteria (16:1ω7c, 18:1ω7c, cy17:0 and cy19:0) plus the actinobacteria markers 10Me16:0, 10Me17:0 and 10Me18:0 were chosen to represent bacterial PLFAs; and the PLFA 18:2ω6c was used to indicate the fungal biomass [45]. Briefly, the total lipidic extract was obtained from 500 mg of each freeze-dried sample by addition of a single-phase extraction mixture, chloroform-methanol-citrate buffer (1∶2∶0.8, *v/v/v*), followed by incubation at 20°C for at least 2 h [46]. The lipid extract was then fractionated into neutral lipids, glycolipids and phospholipids with chloroform (1.5 mL), acetone (6 mL) and methanol (1.5 mL) on silicic acid columns (Bond Elut, Varian Inc., Palo Alto, CA, EEUU). The fraction containing phospholipids was subjected to alkaline methanolysis [47] to obtain the fatty acid methyl esters (FAMEs) after addition of 50 µL of the methyl nonadecanoate FAME (19∶0 at 23 µg mL^−1^) as the internal standard. The extracts containing FAMEs were analysed on a Hewlett-Packard 5890 gas chromatograph (Palo Alto, CA, EEUU) equipped with a flame ionization detector.

The bacterial growth rate was estimated by the leucine incorporation technique [48], as modified by Bååth *et al.* [49]. One gram of each sample (fresh weight) and 20 mL of distilled water were placed in 50-mL centrifuge tubes, shaken for 3 min on a vortex at high speed and centrifuged at 1000× g for 10 min. An aliquot (1.5-mL) of each supernatant (bacterial suspension) was then placed in microcentrifuge tubes and incubated for 2 h at 20°C after adding L-[4,5-^3^H]-leucine (171 Ci mmoL^−1^, 1.0 mCi mL^1^, Amersham) and non-radiactive leucine, to give a final concentration of 270 nM leucine. The incubation was stopped and the macromolecules were precipitated by adding 75 µL cold 100% trichloroacetic acid (TCA). Washing and preparation for scintillation counting were according to Bååth *et al.* [49].

The fungal growth rate was assessed by the acetate in ergosterol method [50] adapted for soil [51]. Briefly, 1 g of each sample (fresh weight) was transferred to test-tubes to which 0.025 mL 1,2-[^14^C] acetic acid (sodium salt, 2.04 GBq mmol^−1^, 7.4 MBq mL^−1^, Amersham), 0.475mL 1mM unlabelled acetate (pH = 6) and 1.5mL distilled water were added, resulting in a final acetate concentration of 0.22 mM acetate. The resulting slurry was incubated at room temperature (22°C) for 8 h, after which 1mL 5% formalin was added to terminate growth. Ergosterol was then extracted, separated and quantified using HPLC and a UV detector (282 nm), according to Rousk & Bååth [52]. The ergosterol peak was collected and the amount of incorporated radioactivity was determined in a liquid scintillation counter.

Total microbial activity was assessed as basal respiration, by measuring the rate of evolution of CO_2_. The samples (2 g, fresh weight) were placed in respiration vials, sealed and incubated at room temperature for 14 h. The amount of CO_2_ produced was then determined by gas chromatography.

### Statistical analysis

Data were analysed by ANOVA, with the type of manure (cow, horse and rabbit) and earthworm treatment (presence and absence) as the main factors. A discriminant function analysis was used to analyse the PLFAs identified in the three different raw manures in order to test whether they were clearly differentiated from each other with respect to the structure of their microbial communities. We also performed a discriminant analysis with the PLFAs identified in the different animal manures after being processed by each earthworm species for one month so as to assess the overall differences in the microbial community structure of the three types of animal manure as a function of the earthworm species. The central concept of the discriminant analysis is to find an equation that classifies new samples into defined groups. The number of dimensions needed to distinguish among the treatments is identified by the number of significant canonical discriminant functions. In the present study, each function is a linear combination of PLFA variables, and the first function has the most power to discriminate among the treatments. The discriminant scores were analysed by ANOVA, as above. The normality and the variance homogeneity of the data were tested prior to ANOVA and discriminant analysis. All statistical analyses were performed with the Statistica software (version 7).
